# Successful Treatment of MEK Inhibitor-Induced Paronychia in Neurofibromatosis with Photodynamic Therapy: A Case Report and Review of the Therapeutic Options

**DOI:** 10.3390/jcm14041104

**Published:** 2025-02-09

**Authors:** Francesca Ambrogio, Teresa Perillo, Domenico Bonamonte, Aurora De Marco, Benedetta Tirone, Carmelo Laface, Gerardo Cazzato, Caterina Foti, Edoardo Mortato

**Affiliations:** 1 Section of Dermatology and Venereology, Department of Precision and Regenerative Medicine and Ionian Area (DiMePRe-J), University of Bari “Aldo Moro”, 70124 Bari, Italy; domenico.bonamonte@uniba.it (D.B.); aurorademarco94@gmail.com (A.D.M.); benedetta.ti96@gmail.com (B.T.); caterina.foti@uniba.it (C.F.); edoardo.mortato@gmail.com (E.M.); 2 Pediatric Hematology-Oncology Division, Department of Pediatrics, University of Bari, 70126 Bari, Italy; terryperillo@hotmail.com; 3 Medical Oncology, Azienda Ospedaliera S.Croce e Carle, 12100 Cuneo, Italy; carmelo.laface@gmail.com; 4 Section of Molecular Pathology, Department of Precision and Regenerative Medicine and Ionian Area (DiMePRe-J), University of Bari “Aldo Moro”, 70124 Bari, Italy; gerardo.cazzato@uniba.it

**Keywords:** Selumetinib, MEK inhibitor, neurofibromatosis, paronychia, nail, photodynamic therapy, pdt

## Abstract

**Background/Objectives:** Selumetinib, a MEK1/2 inhibitor, is commonly used for treating neurofibromatosis type 1 (NF1) and is associated with cutaneous side effects such as paronychia and periungual granulomas. These complications can be painful and difficult to manage, often leading to the discontinuation of treatment. The objective of this study was to evaluate the effectiveness of photodynamic therapy (PDT) as a novel treatment for MEKi-induced paronychia in a patient with NF1. **Methods:** We present a case report of an 18-year-old patient with NF1 who developed painful periungual granulomas on the toenails after 12 months of Selumetinib therapy. PDT was administered using methyl aminolevulinate (METVIX^®^) as the photosensitizing agent, followed by treatment with a red LED light source (630 nm, 37 J/cm^2^ for 8 min and 30 s). The patient was followed up for two months post-treatment and then at two years. **Results:** After a single PDT session, the patient exhibited complete clinical remission of the periungual granulomas and associated pain. No recurrence of the lesions was noted during the two-year follow-up. The patient tolerated the procedure well, reporting only mild discomfort during treatment. **Conclusions:** PDT appears to be an effective, minimally invasive treatment for Selumetinib-induced paronychia and periungual granulomas. This case demonstrates that PDT can provide a complete resolution of symptoms with a single treatment session, offering an alternative to more invasive procedures. Further studies with larger cohorts are needed to establish PDT as a standard treatment option for this condition.

## 1. Introduction

Systemic anticancer drugs often induce nail changes, causing pain and a reduced quality of life [1]. Periungual complications, such as paronychia and pyogenic granulomas, are common, particularly in patients receiving EGFR or MEK inhibitors [2,3,4]. The pathophysiology of MEKi-induced paronychia remains unclear, although it is hypothesized that MAPK inhibition halts keratinocyte proliferation and increases apoptosis, leading to epidermal thinning. This makes the paronychium more susceptible to trauma, which, over weeks to months after drug initiation, triggers inflammation [5]. As a result, the thinning periungual epidermis may eventually become perforated by the lateral edges or corners of the nail plate, leading to a secondary inflammatory response, similar to the presence of a foreign body, and the formation of granulation tissue [6]. Chemotherapy-induced paronychia is typically graded according to the National Cancer Institute (NCI) Common Terminology Criteria for Adverse Events (CTCAE). Sometimes, the reaction is so strong that it causes the formation of pyogenic granulomas-like lesions that are localized in the proximal or lateral nail folds, presenting as painful, smooth, sessile or pedunculated, red, rapidly growing, and often bleeding lesions [4]. A recent literature review identified several treatments for drug-induced paronychia and pyogenic granulomas, including topical beta-blockers, corticosteroids with occlusive dressings, antibiotics, surgical removal, phenolization, and laser therapy. The outcomes vary depending on the number of nails that are affected and the underlying cause [7,8]. However, different approaches, such as photodynamic therapy (PDT), are also evaluated for treating periungual pyogenic granuloma-like lesions during chemotherapy [9]. We present the first reported case of a patient with neurofibromatosis type I (NF1) undergoing Selumetinib therapy who subsequently developed periungual granulomas on the toenails, which were successfully treated with a single session of PDT.

## 2. Case Report

**Figure 1:**
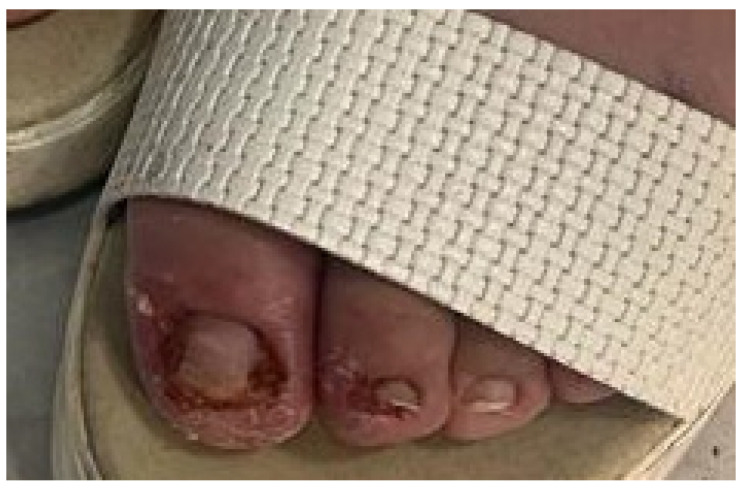
Paronychia with pyogenic periungual granulomas after Selumetinib treatment for neurofibromatosis.

**Figure 2:**
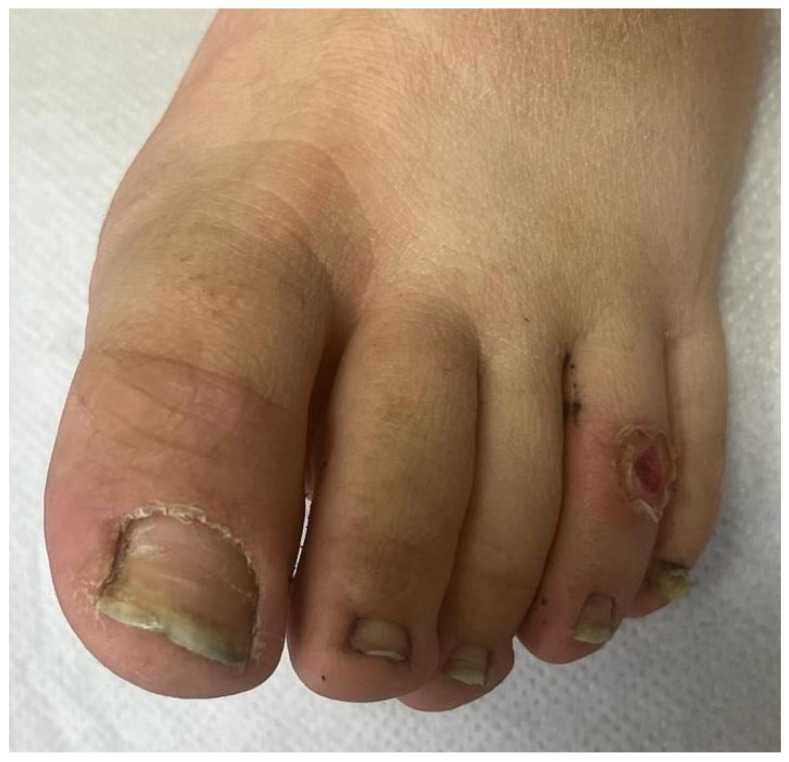
After one session of PDT, the lesions were completely remitted.

Our case concerns an 18-year-old patient with neurofibromatosis type 1, who had been treated with Selumetinib 50 mg daily since November 2021 for severe rachialgia and urinary incontinence due to plexiform neurofibromas of the spinal nerve roots. This medication was prescribed due to the inoperability of the plexiform neurofibromas, which were limiting the patient’s quality of life, both due to pain and daily urinary urgency. In November 2022, the patient presented with the onset of severe paronychia, characterized by erythematous, tender, and fragile granulation tissue beneath the proximal and lateral nail folds of the second and third toes on the right foot, as well as the first toe on the left foot (Figure 1). The patient denied any history of trauma or similar episodes prior to starting Selumetinib therapy. The suspected diagnosis was paronychia with pyogenic periungual granulomas, which was supported by several reported cases in the literature and the timing of onset following Selumetinib administration [8]. The patient reported the onset of these lesions approximately nine months after starting the drug. The patient’s reluctance to undergo topical treatments or nail plate avulsion excluded these conventional options. Considering the potential complications related to neurofibromas, and in consultation with the treating oncologist, it was decided to continue Selumetinib therapy while exploring alternative approaches. Considering the documented success of PDT in similar cases [9], we opted for a session of photodynamic therapy. PDT involved the application of the photosensitizing agent methyl aminolevulinate (METVIX^®^ cream, Galderma Medical Solutions) to the affected area, followed by occlusion for three hours. Subsequently, the area was illuminated using a red 630 nm light-emitting diode (LED) lamp (Aktilite CL128^®^, Galderma, Uppsala, Sweden) at a dosage of 37 J/cm^2^ for 8 min and 30 s. The patient reported only a mild burning sensation during the procedure. At the two-month follow-up, the patient exhibited complete remission of the lesions (Figure 2) and a notable resolution of painful symptoms, indicating a favorable response to the PDT treatment. Subsequently, the patient reported the appearance of other lesions, such as periungual granulomas on the hands, which were also treated with a PDT session, with remission of the clinical picture. At the 2-year follow-up, no recurrence was observed in the patient.

## 3. Discussion

Selumetinib is an oral selective MEK1/2 inhibitor that is approved in the United States (US), the European Union (EU), and other countries for the treatment of pediatric patients (aged ≥2 years in the US and ≥3 years in the EU) with NF1 who have symptomatic, inoperable plexiform neurofibromas [10]. According to a recent multicenter study in children who were treated with Selumetinib, the incidence of paronychia ranges from 31.6% to 51.2% [11]. In the SPRINT phase II study of Selumetinib, paronychia was reported in 23 out of the 50 (46%) patients, with a median treatment time until maximum grade paronychia of 306 days. The long-term use of Selumetinib maintains the paronychia trigger, which frequently results in inoperable and resistant lesions that may require stopping treatment. Timely handling of these side effects may prevent needless treatment pauses. The clinical characteristics and management of Selumetinib-induced paronychia or periungual granulomas remain poorly documented in the evidence-based literature, with treatment often being guided by physician experience and case reports [8]. The treatments that are presented in the literature for this reaction to Selumetinib are similar to those that are reported for periungual reactions due to older drugs (EGFR inhibitors) [9]. Treatments applied were topical agents including β-blockers (e.g., timolol 0.5–1% gel or cream), corticosteroids alone or in combination with gentamycin, brimonidine, oral antibiotics with anti-inflammatory effects (such as doxycycline [12], minocycline, and azithromycin), injectable 10% triamcinolone acetonide, partial nail avulsion, and 80% trichloroacetic acid matricectomy [8]. In the cases of partial or no response, the patients opted for treatment discontinuation or a reduction in the Selumetinib dose due to the pain of the procedures or when they considered a partial improvement to be a satisfying result. It was also noted that the treatments needed to be maintained for a long time [1–6 months], and despite everything, many patients ended up with chemical matricectomy [8]. Topical therapies play a key role in managing Selumetinib-induced paronychia, as highlighted in Palmeiro et al.’s study [8]. However, in most cases (86.7%), further treatments will be required at some point during the patient’s follow-up. Very often, systemic antibiotics have also been used for their anti-inflammatory properties. However, paronychia is unlikely to have an infectious etiology, and the presence of bacteria such as Staphylococcus aureus is typically due to secondary superinfections. However, when superinfection is suspected, culture-guided treatment is recommended. To the best of our knowledge, no cases of paronychia caused by Selumetinib have been treated with PDT. PDT is a clinically approved and minimally invasive therapeutic procedure that exploits a substance called photosensitizer and a light source to exercise a selective cytotoxic activity [13]. The photosensitizer is a molecule that is mainly absorbed by cells with increased metabolic activity and will cause oxidative damage in infected cells once the light ray irradiates the target area of the skin. It must be applied to the lesions for a certain amount of time before irradiating the area to ensure its efficacy. Along with the photosensitizer, the irradiation time from the light source must be accurately set to deliver a specific energy load. PDT is currently indicated for treating actinic keratosis, basal cell carcinoma, and squamous cell carcinoma in situ (Bowen’s disease), as well as numerous inflammatory conditions [13]. Moreover, some studies have demonstrated that endothelial cells generate protoporphyrin IX from photosensitizers, and PDT can destroy vascular endothelial cells in vitro and in vivo [14]. Based on these considerations, some authors recently proposed PDT for treating pyogenic periungual granulomas that were induced during anti-EGFR therapy [9]. The authors of the referenced study suggested that at least three sessions of photodynamic therapy (PDT), spaced 20 days apart, were necessary to achieve complete remission of lesions in two patients and significant improvements in the others [9]. In contrast, in our clinical case, only one session of PDT was required to achieve complete remission of pyogenic periungual granulomas, highlighting a significant difference in the number of sessions that were needed.

We did not use the same method as the previous group [9], because they applied 5-methyl aminolevulinate acid for one hour, and the area was irradiated for 10 min. However, the improved clinical response in only one session in our case is also likely explained by the recent finding that systemic MEK inhibition, such as Selumetinib, enhances the efficacy of 5-aminolevulinic acid-photodynamic therapy [15]. To our knowledge, our clinical case appears to be the first experience of the successful treatment of paronychia and periungual pyogenic granulomas that were induced by therapy with Selumetinib with a single session of PDT. The positive response of these drug reactions to PDT suggests that it may avoid the need for repeated topical treatments, systemic antibiotics, and even destructive nail interventions. PDT’s effectiveness and tolerability make it a promising therapeutic approach for treating this drug reaction. However, our data need to be applied to a larger cohort of patients to establish a standardized treatment protocol.

## Data Availability

The data presented in this study are available on request from the corresponding author due to privacy reasons.
